# Laparoscopic Intraperitoneal Onlay Mesh Plus Repair of a Hernia Adjacent to a Vesicocutaneous Urinary Diversion: A Case Report

**DOI:** 10.70352/scrj.cr.26-0323

**Published:** 2026-06-24

**Authors:** Takahiko Omameuda, Masaru Koizumi, Makiko Tahara, Yoshiyuki Inoue, Hiroyuki Kitabayashi, Atsushi Miki, Mikio Shiozawa

**Affiliations:** 1 Department of Surgery, Tochigi Medical Center, Tochigi, Tochigi, Japan; 2 Division of Gastroenterological, General, and Transplant Surgery, Department of Surgery, Jichi Medical University, Shimotsuke, Tochigi, Japan

**Keywords:** abdominal wall hernia, intraperitoneal onlay mesh plus technique, vesicocutaneous fistula

## Abstract

**INTRODUCTION:**

Hernias occurring adjacent to a urinary diversion are extremely rare, and their classification and optimal surgical management have not yet been established.

**CASE PRESENTATION:**

A 76-year-old man presented with a hernia adjacent to a vesicocutaneous urinary diversion after undergoing laparoscopic abdominoperineal resection for rectal cancer combined with robot-assisted radical prostatectomy, resulting in sigmoid colostomy and vesicocutaneous fistula formation. Laparoscopic repair was performed using the intraperitoneal onlay mesh (IPOM) plus technique. After primary fascial closure, a trimmed composite mesh was placed with adequate overlap and fixed using a modified fixation strategy combining non-absorbable tacks and absorbable sutures. Tacking was deliberately avoided in the anatomically hazardous region adjacent to the external iliac vessels, and fixation in this area was achieved using superficial sutures only. The postoperative course was uneventful, with no recurrence or mesh-related complications during the 18-month follow-up.

**CONCLUSION:**

Laparoscopic IPOM plus repair may be a feasible option for hernias adjacent to a vesicocutaneous urinary diversion when primary fascial closure and safe mesh fixation can be achieved with careful consideration of pelvic anatomy and adjacent neurovascular structures. However, further accumulation of cases and long-term follow-up are required to establish its safety.

## Abbreviations

IPOM
: intraperitoneal onlay mesh
PSH
: parastomal hernia

## INTRODUCTION

PSH is defined as the protrusion of contents of the abdominal cavity through the abdominal wall in the direct proximity of a stoma.^1)^ In the field of urostomy, PSH has been relatively frequently reported following ileal conduit urinary diversion. In contrast, a hernia developing adjacent to a vesicocutaneous fistula is extremely rare, and its classification as either a PSH or an incisional hernia remains controversial. We searched the PubMed database (National Library of Medicine, Bethesda, MD, USA) using the keywords “hernia” and “vesicocutaneous fistula” for articles published between 1980 and December 2025. Although several reports have described incisional hernia after tube cystostomy creation as similar lesions,^2–5)^ none involved a hernia adjacent to a vesicocutaneous urinary diversion. Therefore, the clinical characteristics and optimal management of this condition remain unclear.

Laparoscopic hernia repair offers several advantages, including procedural safety, faster postoperative recovery, fewer complications, and lower recurrence rates.^6)^ The laparoscopic IPOM technique is a widely used mesh-based approach for abdominal wall hernia repair.^7)^ The laparoscopic IPOM plus technique further reinforces this repair by suturing the fascial defect prior to mesh placement and is currently recommended by the International Endohernia Society.^8)^ Herein, we report a rare case of a hernia adjacent to a vesicocutaneous urinary diversion that was successfully repaired using the laparoscopic IPOM plus technique, with particular attention to anatomical safety considerations.

## CASE PRESENTATION

A 76-year-old man presented to the Department of Digestive Surgery with abdominal pain concentrated near a vesicocutaneous fistula. His medical history was notable for prostate cancer, for which he had undergone radiotherapy at the age of 67 years. The total radiation dose was 78 Gy (**Fig. 1A**). At the age of 74 years, he underwent laparoscopic abdominoperineal resection for rectal cancer with invasion of the prostate, along with sigmoid colostomy combined with robot-assisted radical prostatectomy. At the time of this surgery, a vesicocutaneous fistula was intentionally created as a salvage urinary diversion, as prior radiotherapy was considered to confer a high risk for urethral anastomosis. Urinary diversion was established using the Blocksom technique. The bladder was exteriorized through the right rectus abdominis muscle and fixed to the abdominal wall to enable continuous urinary drainage. He was found to have developed a hernia adjacent to the vesicocutaneous urinary diversion (**Fig. 1B**), which was associated with localized pain 18 months after the initial surgery. Therefore, surgical repair was indicated, and the operation was performed 3 months after the diagnosis. No PSH was observed at the sigmoid colostomy site. The hernia defect was anatomically distinct from the previous port sites, making a port-site hernia unlikely. Given the patient’s history of pelvic radiotherapy, radiation-induced tissue fragility may have contributed to fistula formation, although the exact mechanism could not be definitively determined. The laparoscopic approach was selected because it enables superior assessment of the intraperitoneal cavity and has a relatively low risk of mesh contamination.

**Fig. 1:**
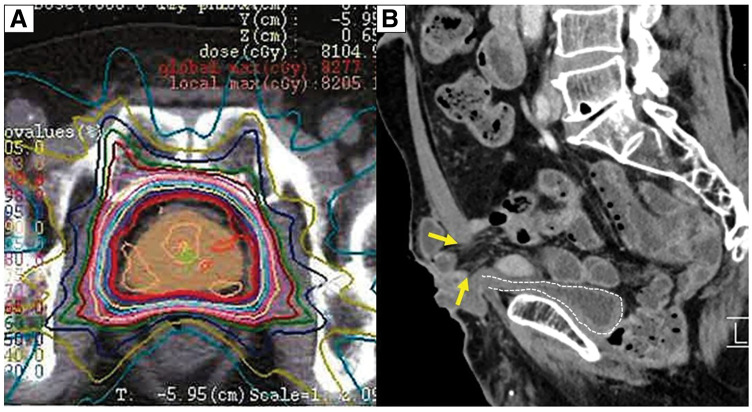
Preoperative imaging findings. (**A**) CT image (axial section) showing the actual irradiation field for prostate cancer radiation therapy. (**B**) Contrast-enhanced abdominal CT (sagittal section) demonstrating a hernia (yellow arrows) adjacent to a vesicocutaneous urinary diversion (yellow arrows). The white dotted line indicates the urinary bladder.

The surgery was conducted laparoscopically using a 4-port technique, which involved placing a camera trocar supraumbilically and inserting 3 additional trocars: 1 in the epigastrium and 2 in the right upper abdomen (**Fig. 2A**). Intraoperatively, the urinary bladder was elevated extracorporeally through the right rectus abdominis muscle, and a hernia defect measuring approximately 3.0 × 2.0 cm was identified on the cranial side of the bladder. No significant adhesions were observed between the bladder and the surrounding organs. The left medial inguinal fossa appeared fragile with a mild depression (**Fig. 2B**). We closed the fascial defect using a non-absorbable barbed suture, 1-0 V-Loc PBT (Medtronic, Minneapolis, MN, USA) (**Fig. 2C**). The surgical strategy was to cover the stoma site, the fascial closure area, and the left medial inguinal fossa with a mesh, providing at least a 5-cm overlap over the normal abdominal wall. An oval Symbotex Composite Mesh (Medtronic) was trimmed from 12 × 20 to 12 × 15 cm by removing 2.5 cm from each lateral side prior to placement. After trimming, the mesh was reshaped into a rounded trapezoidal configuration to provide adequate overlap around the urinary diversion site while avoiding excessive folding. Following primary fascial closure, circumferential tacking secured the mesh. Non-absorbable tacks were used for fixation to the abdominal wall, while absorbable sutures were selectively used in proximity to the bladder to minimize the risk of injury. Tacks were deliberately avoided in the anatomically hazardous region adjacent to the external iliac vessels. In the region adjacent to the external iliac vessels, fixation was limited to superficial suturing of the peritoneum with careful attention paid to avoiding injury to critical neurovascular structures and without penetrating the underlying fascia or vascular structures. On the dorsal side, to enhance mesh stabilization, the mesh was additionally secured to the peritoneum adjacent to the left external iliac vessels and bladder wall using an absorbable braided suture (3-0 Vicryl, polyglactin 910; Johnson & Johnson MedTech, Somerville, NJ, USA) (**Fig. 2D** and **2E**). Although the repair shared certain technical similarities with the Sugarbaker approach in PSH repair, including lateralized mesh placement around the urinary diversion, no lateralization of the intra-abdominal viscera or creation of a lateral tunnel was required because the urinary diversion consisted of a vesicocutaneous fistula rather than an intestinal stoma. Therefore, the procedure was considered a modified laparoscopic IPOM plus repair with technical similarities to the Sugarbaker approach. The postoperative course was uneventful; neither recurrence nor mesh-related complications nor postoperative neurological symptoms, including groin numbness, thigh pain, or dysesthesia, were observed or reported by the patient during the 18-month follow-up period (**Fig. 3**).

**Fig. 2:**
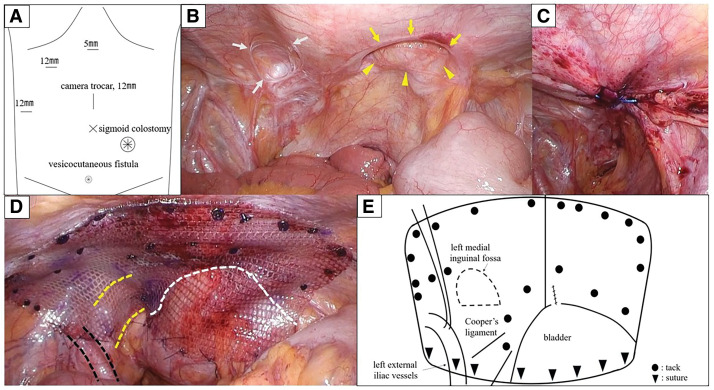
Intraoperative findings. (**A**) Schematic explanation of the trocar placement. (**B**) The hernia defect (yellow arrows) measuring approximately 3.0 × 2.0 cm was identified on the cranial side of the bladder (yellow arrowheads). The left medial inguinal fossa appeared fragile with mild depression (white arrows). (**C**) Closure of the fascial defect using a non-absorbable barbed suture. (**D**) Mesh fixation technique. Non-absorbable tacks were used for fixation to the abdominal wall, while absorbable sutures were selectively used in proximity to the bladder to minimize the risk of injury. Tacks were deliberately avoided in the anatomically hazardous region adjacent to the external iliac vessels. In the region adjacent to the external iliac vessels, fixation was limited to superficial suturing of the peritoneum, with careful attention to avoid injury to critical neurovascular structures and without penetration of the underlying fascia or vascular structures. On the dorsal side, to enhance mesh stabilization, the mesh was additionally secured to the peritoneum adjacent to the left external iliac vessels and the bladder wall using absorbable braided sutures. (**E**) Schematic of the mesh fixation.

**Fig. 3:**
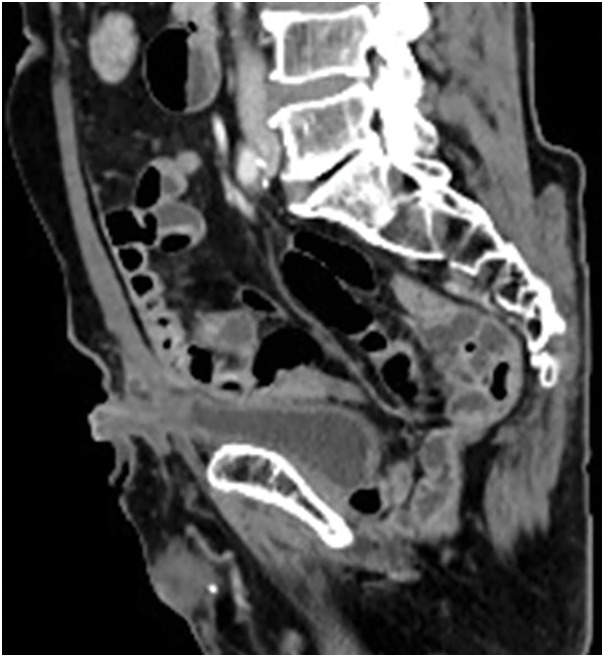
Postoperative imaging findings. Follow-up contrast-enhanced abdominal CT (sagittal section) obtained 18 months after repair demonstrated no evidence of hernia recurrence.

## DISCUSSION

This case involved a rare hernia adjacent to a vesicocutaneous fistula that had been intentionally created as a urinary diversion and was successfully managed using the laparoscopic IPOM plus technique. The lesion developed adjacent to the vesicocutaneous fistula used for urinary diversion and shared certain anatomical features of a PSH, as it occurred through the abdominal wall in the direct proximity of a stoma-like structure. However, it was considered to more closely resemble an incisional hernia because of the prior surgical manipulation and the possible contribution of radiation-related tissue fragility. Therefore, we considered the term “hernia adjacent to a vesicocutaneous urinary diversion” to be more appropriate. A narrative review of previously reported related cases yielded 3 important clinical insights.

First, to the best of our knowledge, no previous reports have described a hernia adjacent to a vesicocutaneous urinary diversion, although several previous reports have described incisional hernia following tube cystostomy creation.^2–5)^ This makes the present case clinically unique. Therefore, surgeons should be aware of this rare but potentially late complication, particularly in patients undergoing complex pelvic procedures.

Second, the laparoscopic IPOM plus technique may be a feasible option for treating abdominal wall hernias adjacent to urinary diversion in selected cases. Although the present lesion may not represent a classic PSH, several technical concepts used in laparoscopic PSH repair were considered applicable in this case. The 2018 European Hernia Society guidelines discourage suture-only repair for elective PSH surgery because of the high risk of recurrence; however, a definitive consensus regarding the optimal surgical approach has not yet been established.^9)^ In the present case, laparoscopic IPOMplus repair was selected because primary fascial closure could be achieved without excessive tension, as no adhesions were present within or around the hernia defect. Accordingly, suitable candidates for this approach may include patients in whom secure fascial closure can be achieved laparoscopically, contamination is absent, and sufficient distance from critical pelvic neurovascular structures can be maintained. If secure fascial closure had not been technically feasible in this case, the conventional IPOM technique would have been considered as an alternative. A critical consideration in this case is the safety of mesh fixation in anatomically sensitive regions. In particular, the region adjacent to the external iliac vessels contains critical neurovascular structures, and deep fixation in this area should be avoided whenever possible. In the present case, no tacks were placed within this region, and fixation in proximity to the external iliac vessels was limited to superficial suturing of the peritoneum, with careful attention to avoid injury to critical neurovascular structures. Although no neurological symptoms were reported by the patient, the absence of a systematic neurological assessment represents a limitation of this report.

Third, a thorough discussion on the safety of fixing the bladder with a mesh is required. Symbotex Composite Mesh is a novel 3D monofilament polyethylene terephthalate textile, the visceral surface of which is coated with a modified absorbable hydrophilic film composed of a mixture of collagen and glycerol. This protective hydrophilic film remains intact on the visceral side and serves as the sole component that is in direct contact with the intra-abdominal viscera, thereby limiting the potential for adhesion formation.^10)^ In the present case, the bladder was positioned on the abdominal wall side of the mesh, whereas the intra-abdominal viscera, including the bowel, were located on the visceral side. Therefore, a composite mesh with an anti-adhesive barrier on the visceral surface was considered appropriate to minimize the risk of adhesion to the intra-abdominal organs and erosion of the bladder wall. When securing the mesh to the bladder, absorbable braided sutures were placed superficially on the bladder wall to avoid full-thickness penetration. Careful consideration was given to maintaining minimal mesh tension and restricting the number of fixation points, thereby reducing the risk of bladder perforation and subsequent mesh erosion. Additionally, to minimize the risk of mesh-related infection, surgery was performed in the absence of active infection or urinary leakage. However, direct evidence supporting the safety of mesh fixation to the bladder wall remains limited, and this approach should be interpreted with caution and considered on a case-by-case basis. Moreover, although no mesh-related complications were observed during the 18-month follow-up period, late complications such as mesh erosion, infection, and fistula formation may occur beyond this period.

This report has several limitations. First, it is based on a single case. Second, the follow-up period of 18 months is insufficient to fully evaluate long-term outcomes and late complications. Third, neurological assessment was not systematically performed, which limits the evaluation of safety in relation to fixation near critical anatomical structures.

## CONCLUSIONS

The laparoscopic IPOM plus technique may be feasible in carefully selected cases of hernia adjacent to a vesicocutaneous urinary diversion. When surgeons possess detailed pelvic anatomical knowledge and carefully consider neurovascular safety, mesh placement around a vesicocutaneous fistula may be technically feasible. However, given the anatomical risks and the limited follow-up in this single case, further evidence is needed to establish the safety and durability of this approach. Further accumulation of cases and long-term follow-up are required.

## DECLARATIONS

### Availability of data and materials

All data supporting the conclusions of this study are included in this published article.
